# External application of herbal medicine for gout: A protocol for systematic review and meta-analysis of randomized controlled trials

**DOI:** 10.1097/MD.0000000000025649

**Published:** 2021-04-23

**Authors:** Ji Hye Hwang, Ho Sueb Song

**Affiliations:** Department of Acupuncture & Moxibustion Medicine, College of Korean Medicine, Gachon University, Seongnam, Republic of Korea.

**Keywords:** external application, gout, herbal medicines, hyperuricemia, protocol, systematic review

## Abstract

**Background:**

Gout affects a significant portion of the population worldwide annually. Numerous studies have been reported mainly in East Asia, explaining the use of traditional herbal decoctions for gout treatment. Our systematic review will be conducted to critically evaluate the evidence for the safety and effectiveness of external applications of herbal medicines on gout.

**Methods:**

Two independent researchers will perform electronic literature searches, study selection, data extraction, and quality assessment. To identify randomized controlled trials (RCTs) involving various external applications of herbal medicine for gout, a search will be carried out using the following 7 electronic databases: MEDLINE, EMBASE, Cochrane Library, KoreaMed, Oriental Medicine Advanced Searching Integrated System, Korean Studies Information Service System, and China National Knowledge Infrastructure. Each electronic database will be searched for articles published from their inception to the present date. Studies will be selected based on predefined criteria and summarized data regarding study participants, interventions, control groups, outcome measures, side effects, and risk of bias. There are no restrictions on publication status or language. Studies that evaluated any type of external application of herbal medicines will be eligible for inclusion, and the primary outcome will be the blood uric acid level. The methodological quality of the included RCTs will be assessed using the Cochrane risk-of-bias tool.

**Results:**

The present study will evaluate effectiveness and safety of external application of herbal medicines for gout.

**Conclusion:**

Our findings will establish evidence for the external application of herbal medicines for gout and will be informative for patients with gout, clinicians, policymakers, and researchers.

The results of this systematic review will be published in a peer-reviewed journal and disseminated electronically and in print. This review will be updated to inform and guide healthcare practices.

## 1 Introduction

Gout with clinical symptoms, including tophi deposition, characteristic recurrent acute arthritis, chronic gouty arthritis, and deformed joints,^[1–2]^ often affects the kidneys, causing chronic interstitial nephritis and forming urinary tract stones composed of uric acid, and can cause joint damage or kidney failure in severe cases.^[3]^ Gout affects a significant portion of the population worldwide annually,^[4]^ and according to the estimates of the World Health Organization (WHO), 3.9% of people worldwide are suffering from gout.^[5]^ The high incidence of gout is no longer limited to the elderly, and there is a tendency for early onset in younger populations.^[6]^ The acute onset of arthritis, joint malformations, chronic joint injuries, and the formation of renal calculus not only reduce the quality of life but also lead to disability.^[7]^

Conventional Western medicine treatments for gout focus primarily on the treatment of high uric acid.^[8]^ Drugs such as colchicines, corticosteroids, and nonsteroidal antiinflammatory drugs (NSAIDs) have been used to treat the acute onset of gout.^[9]^ These drugs show good short-term effects, but long-term use can lead to gastrointestinal reactions, rashes, systemic vacuities, and even renal failure.^[10,11]^ In addition, such drugs cannot prevent, halt, or reverse the progression of this complicated disease. Conventional treatments for gout have several side effects and limitations, so more attention should be paid to complementary and alternative medicine (CAM).

In CAM centered in East Asia, there are numerous reports on the use of traditional herbal medicines to treat gout.^[8]^ In a previous systematic review of the clinical efficacy and safety of traditional Western medicine and Chinese herbal decoctions for gout treatment, it was reported that traditional Western medicine and Chinese herbal decoctions showed similar clinical efficacy, but Chinese herbal decoctions were found to be superior to Western medicine in suppressing the side effects of drugs.^[8]^ This systematic review aims to critically evaluate the evidence for its effectiveness and safety, focusing on external application among herbal medicines for gout.

## 2 Methods

### 2.1 Study protocol registration

We will conduct this systematic review report in accordance with the Preferred Reporting Items for Systematic Reviews and Meta-Analyses protocols (PRISMA-P).^[12]^ The protocol was registered in the Research Registry (registration number, reviewregistry1120)

https://www.researchregistry.com/browse-the-registry#registryofsystematicreviewsmeta-analyses/registryofsystematicreviewsmeta-analysesdetails/60554737714b82001edda16e/

### 2.2 Ethics and dissemination

As the study will review the published literature, no ethical approval is required, as there will be no patient recruitment and no personal data collection. The results of this systematic review will be disseminated by publishing a manuscript in a peer-reviewed journal or presenting it at a related conference. Clinical practice guidelines (CPGs) were prepared and disseminated, including this study.

### 2.3 Eligibility criteria

#### 2.3.1 Types of participants

Adult patients (aged ≥18 years) diagnosed with gout will be included, with no restrictions on any other conditions, such as severity of symptoms, sex, country of origin, or education status.

#### 2.3.2 Types of interventions and controls

Both treatment with external application of herbal medicines alone and concurrent treatment with another therapy will be considered acceptable if only external application of herbal medicines is applied to the intervention group and the other treatment is provided equally to both the intervention and control groups. Studies evaluating any form of external application of herbal medicines, such as plaster cream, washing liquid, cream, etc, will be eligible for inclusion.

For control groups, we will consider placebo or sham, no interventions, and any type of control intervention compared with external application of herbal medicines.

#### 2.3.3 Types of studies

Prospective RCTs that evaluate the effectiveness of the external application of herbal medicines for gout will be considered. Non-RCTs, case reports, observational studies, cross-sectional studies, pilot studies, animal studies, surveys, and systematic review protocols will be excluded.

#### 2.3.4 Outcomes and prioritization

The primary outcomes will be assessed using blood uric acid levels and pain scores, such as a visual analog scale or a numeric scale.

Secondary outcomes will include duration of pain relief, total effective rate, inflammatory markers such as erythrocyte sedimentation rate and C-reactive protein, swelling, and incidence of adverse events.

### 2.4 Data sources and search strategy

**Figure 1:**
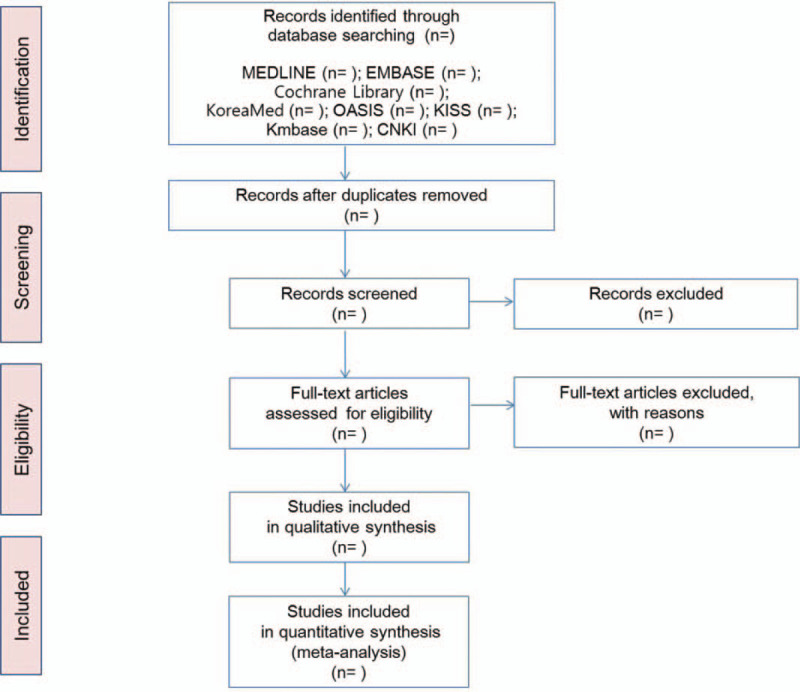
Study flow. PRISMA flow diagram. OASIS = Oriental Medicine Advanced Searching Integrated System, KISS = Korean Studies Information Service System, KMbase = Korean Medical Database, CNKI = China National Knowledge Infrastructure.

Databases and search terms will be determined through discussions between all authors before the literature search is executed. Two independent researchers will perform electronic literature searches, study selection, data extraction, and quality assessment. The following electronic databases will be searched for studies from inception to the present date: Medline, EMBASE, the Cochrane Central Register of Controlled Trials, Oriental Medicine Advanced Searching Integrated System, Korean Studies Information Service System, Research Information Service System, Korean Medical Database, Korea Citation Index, and China National Knowledge Infrastructure. Disagreements between the 2 researchers will be resolved through consensus. There are no restrictions on publication status or language (Fig. 1).

### 2.5 Data extraction

We will review all identified articles to evaluate their eligibility for inclusion. In the case of uncertainties, the authors will be contacted for further information. After the selection of studies, we will extract the following data from the selected articles: author, year of publication, study design, participants (age, sex), diseases or disorders, control intervention, outcome measures, main results, and adverse events. Two reviewers will conduct the data extraction with a recognized data extraction form agreed upon by all reviewers, including author name (s), age, country, year of publication, characteristics of participants, intervention, method of randomization, blinding, control treatment, main outcomes, and adverse events. The reviewers will perform a quality assessment using a predefined data extraction form. Disagreements between the 2 researchers will be resolved through consensus.

### 2.6 Data synthesis and analysis

The differences between the intervention and control groups will be assessed. Mean differences (MDs) with 95% confidence intervals (CIs) will be used to measure the effects of treatment for continuous data. We will convert other types of data into MDs. For outcome variables on different scales, standard MDs with 95% CIs will be used. For dichotomous data, we will present treatment effects as relative risks (RRs) with 95% CIs; other binary data will be converted into RR values. All statistical analyses will be conducted using Cochrane Collaboration software program Review Manager (version 5.3; Copenhagen, The Nordic Cochrane Centre, the Cochrane Collaboration, 2014) for Windows. We will contact the corresponding authors of studies with missing information to obtain and confirm the data, if possible. Where appropriate, we will consolidate data across the study to perform a meta-analysis using fixed or random effects. We will use GRADEpro software from Cochrane systematic reviews to create a summary of findings table. Intention-to-treat analyses, including all randomized patients, will be performed to address missing data. A last observation carry-forward analysis will be performed in cases with missing outcome data. The original source or published trial reports of the data will be reviewed in cases where individual patient data are initially unavailable.

### 2.7 Assessment of risk of bias in individual studies and analysis of subgroups or subsets

We will use the Cochrane Collaboration risk-of-bias tool to assess the risk of bias of the included RCTs. Domains including random sequence generation, allocation concealment, blinding of participants, personnel, and outcome assessors, completeness of data outcome, selective reporting, and other biases will be assessed as “low risk,” “unclear risk,” or “high risk.” We will assess other bias items as the statistical baseline imbalance severity between the treatment and control groups, including the participants’ mean age, sex, disease period, or disease severity. Any disagreement will be resolved through discussion between all authors.

If significant heterogeneity is identified (*P* < .1, χ^2^ test or Higgins *I*^2^ ≥ 50%), a subgroup analysis will be performed.

## 3 Discussion

Herbal medicines in CAM have been developed based on unique theories such as traditional Chinese medicine (TCM) and traditional Korean medicine (TKM) theory and have been used for internal and external treatment of various diseases for thousands of years.^[13,14]^ Numerous studies have been reported mainly in East Asia, explaining the use of traditional herbal decoctions for gout treatment.^[15]^ Herbal products have been offered in a variety of dosage forms, such as decoctions, herbal powders, capsules, tablets, herbal teas, tinctures, alcoholic beverages, herbal soaps, ointments, and creams.^[16]^ External treatment involves the application of drugs to the surface or point of illness.^[17]^ The application of herbal medicine to acupuncture points is used to regulate meridians, yin-yang, and qi-blood through the pharmacological action of herbal medicines and their stimulation of acupuncture points for disease prevention and treatment.^[18]^ External use of herbal medicine in traditional medicine has been regarded as less expensive and safer than conventional medicines, such as oral or topical drugs.^[17,19]^

CAM has been treating gout since ancient times using traditional medicines such as TCM and TKM. In TCM and TKM, gout is classified as a Bi pattern, usually due to qi stagnation in the meridians and collaterals.^[16,20,21]^ In general, manifestations of the Bi pattern are mainly characterized as pain, numbness, and heaviness of muscles, tendons, and joints, or swelling of joints with hot sensation and limitation of movement.^[21,22]^ According to TCM, the primary causes are the deficiency of healthy qi and the invasion of pathogenic factors such as wind, cold, dampness, or heat.^[21,23]^ In recent decades, classical TCM formulas and agents isolated from some Chinese herbal medicines have been applied to treat gout and have achieved satisfactory results.^[16]^ Although there have been reports on the clinical therapeutic effect of the external application of traditional herbal medicines to acute gouty arthritis,^[24–26]^ evidence such as a systematic review focusing on the external application of herbal medicines to gout is lacking.

Therefore, in the present systematic review and meta-analysis, clinical symptoms and laboratory indicators will be analyzed to evaluate evidence regarding the efficacy and safety of external use of herbal medicine in traditional medicine used for gout. We hope that our findings will be useful as a resource to guide optimized clinical treatment strategies for health policymakers, clinical practitioners, patients with gout, and researchers. We expect that the study will be used to establish an integrated model combining both Eastern and Western treatment of gout.

## Author contributions

HSS conceived the study and developed the criteria. JHH and HSS searched the literature and analyzed the data. JHH wrote the protocol, and JHH and HSS revised the manuscript. All authors have read and approved the final manuscript.

**Conceptualization:** Ho Sueb Song.

**Data curation:** jihye hwang.

**Funding acquisition:** Ho Sueb Song.

**Methodology:** jihye hwang.

**Writing – original draft:** jihye hwang.

**Writing – review & editing:** jihye hwang, Ho Sueb Song.
